# From static pathology to dynamic immunity: immunological plasticity and histopathological remodeling in atopic dermatitis and psoriasis

**DOI:** 10.3389/fimmu.2026.1770896

**Published:** 2026-02-13

**Authors:** Wei Shen, Yi Yao, Ying-Ming Ma, Xiao-Lei Xie, Shun-Li Tang, Yuan Wu, Hua-Jie Zhong, Yu-Mei Li, Hui Sun

**Affiliations:** 1 Department of Dermatology, Huzhou Central Hospital, Fifth School of Clinical Medicine of Zhejiang Chinese Medical University, Huzhou, Zhejiang, China; 2 Department of Dermatology, Huzhou Central Hospital, Affiliated Central Hospital of Huzhou University, Huzhou, Zhejiang, China; 3 Institute of Regenerative Medicine, Affiliated Hospital of Jiangsu University, Jiangsu University, Zhenjiang, Jiangsu, China; 4 Department of Dermatology, Affiliated Hospital of Jiangsu University, Jiangsu University, Zhenjiang, Jiangsu, China

**Keywords:** atopic dermatitis, histopathology, immune plasticity, precision medicine, psoriasis

## Abstract

Atopic dermatitis (AD) and psoriasis are the two canonical chronic inflammatory skin disorders, classically differentiated by their distinct histopathological features and immune polarization—Th2-dominant in AD versus IL-23/Th17-dominant in psoriasis. However, this conventional dichotomy is increasingly contested by clinical observations, such as the mixed immunophenotypes seen in Asian AD and overlap syndromes, which complicate clear-cut classification. Growing evidence highlights substantial immunological plasticity and pathological overlap, suggesting that these diseases may represent segments of a continuous spectrum rather than discrete entities. In this Mini Review, we integrate classical morphological observations with recent advances in molecular immunology to explore the mechanisms underlying these tissue responses. We examine how immune cell plasticity—particularly of tissue-resident memory T cells—and their crosstalk with the structural microenvironment contribute to disease heterogeneity and therapy-induced phenotypic shifts. We propose that a shift from static histologic evaluation toward dynamic immunopathological profiling is crucial for narrowing the gap between conventional diagnosis and the emerging paradigm of precision medicine.

## 1 Introduction

Chronic inflammatory skin diseases arise from dysregulation of the delicate immune-stromal network, with AD and psoriasis representing the two most prevalent—yet immunopathologically distinct—clinical paradigms. Historically, these disorders have been classified according to a binary framework: spongiotic dermatitis with dominant Th2 activation typifies AD, whereas psoriasiform hyperplasia driven by IL-23/Th17 axis activation characterizes psoriasis (1). This dichotomous model has successfully informed the development of targeted biologics, transforming therapeutic landscapes and significantly improving patient quality of life. Nevertheless, a substantial “precision medicine gap” persists (2), as a growing number of clinical observations contest this rigid separation. Certain patient subsets exhibit therapeutic recalcitrance to pathway-specific agents, while others develop paradoxical reactions—such as the emergence of psoriasis-like lesions during anti-IL-4/13 therapy—suggesting that blocking one immune axis may inadvertently unmask or amplify another (3).

This clinical complexity is mirrored histopathologically. Mixed or hybrid patterns are increasingly recognized, particularly in specific ethnic populations, such as the Asian AD phenotype, which frequently demonstrates psoriasiform epidermal hyperplasia and Th17 activation. Similarly, in chronic stages of either disease, traditional morphological distinctions often blur, further challenging diagnostic boundaries (4, 5). These observations collectively suggest that AD and psoriasis are not static or mutually exclusive entities, but rather reflect a dynamic spectrum of inflammation shaped by underlying immunological plasticity. These observations collectively suggest that AD and psoriasis represent dominant states along a continuous inflammatory spectrum (summarized in **Supplementary Table 1**).

In this Mini Review, we synthesize contemporary insights into the immune and pathological architecture of AD and psoriasiform dermatoses, with a particular focus on the mechanisms of immune plasticity. We examine how overlaps and transitions between disease states occur, emphasizing the role of tissue-resident immune cells and stromal components in phenotypic shifting. We propose that moving beyond static histological classification toward integrated, dynamic immune-pathological profiling is essential for bridging the current diagnostic and therapeutic gaps, ultimately paving the way for truly personalized management strategies (6).

## 2 Cutaneous immune homeostasis and pathological disruption

### 2.1 Normal skin immune architecture

Under physiological conditions, the skin functions as a highly integrated immune−stromal ecosystem. Keratinocytes and fibroblasts are now recognized not merely as structural elements but as active immunomodulators: they continuously maintain barrier integrity and dynamically shape the local microenvironment through complex signaling networks (7, 8). A spatially organized network of resident immune cells—including Langerhans cells and tissue-resident memory T cells (TRM)—is essential for maintaining immunological vigilance. Recent single−cell transcriptomic atlases further reveal that these populations are regulated by intrinsic homeostatic checkpoints, which maintain a state of “alert quiescence.” This finely tuned balance enables potent immunosurveillance while effectively restraining autoinflammatory responses (9, 10).

### 2.2 From homeostasis to inflammation: pathological remodeling

Pathology arises when genetic predispositions or environmental triggers disrupt this homeostatic equilibrium. The process often begins with a dysregulation in epithelial−immune crosstalk, in which keratinocytes release alarmins (e.g., TSLP, IL−33), initiating self−amplifying inflammatory circuits (11). Sustained inflammatory signaling drives profound tissue remodeling. Structural components undergo maladaptive changes, such as aberrant differentiation and hyperplasia, while immune populations shift toward pathogenic phenotypes. Histopathologically, these alterations are classically identified as spongiotic or psoriasiform patterns. However, emerging genomic evidence suggests that these patterns represent dynamic transitions along a continuous inflammatory spectrum, rather than discrete endpoints, highlighting the remarkable plasticity of the cutaneous immune response (6, 12).

## 3 Atopic dermatitis: immune–pathological characteristics

### 3.1 Classical pathological features

**Figure 1:**
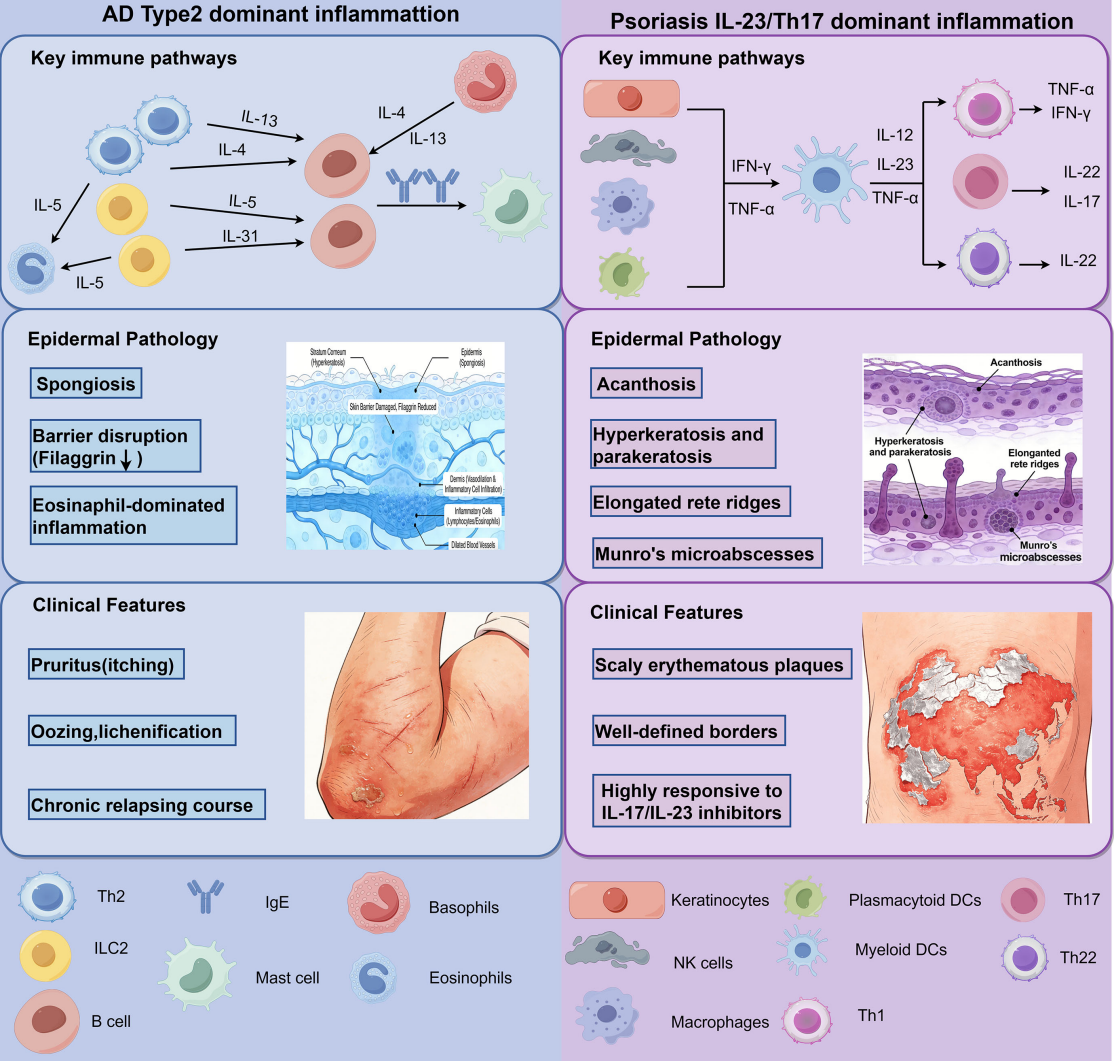
Classical immune-pathological dichotomy between atopic dermatitis and psoriasis.

The hallmark histopathological feature of acute AD is spongiosis, representing intercellular edema within the epidermis. This change is frequently accompanied by exocytosis of lymphocytes and, in certain cases, the formation of intraepidermal vesicles. The epidermis often displays variable degrees of acanthosis, while the stratum corneum may exhibit parakeratosis or compact hyperkeratosis, depending on disease chronicity and anatomical location (**Figure 1**) (13, 14).

In the dermis, a superficial perivascular inflammatory infiltrate is commonly observed, predominantly consisting of T lymphocytes, with variable contributions from eosinophils, mast cells, and antigen-presenting cells. Dilated capillaries and dermal edema are frequent in acute lesions. In chronic AD, repeated inflammatory insults lead to epidermal hyperplasia, hyperkeratosis, and fibrotic changes within the papillary dermis, often accompanied by a reduction in overt spongiosis. These chronic changes can obscure classical features and complicate histopathological diagnosis, complicating the interpretation of biopsy samples (15, 16). In the dermis, a superficial perivascular inflammatory infiltrate is commonly observed, composed predominantly of T lymphocytes, with variable contributions from eosinophils, mast cells, and antigen-presenting cells. Dilated capillaries and dermal edema are frequent in acute lesions. In chronic AD, repeated inflammatory insults lead to epidermal hyperplasia, hyperkeratosis, and fibrotic changes within the papillary dermis, often accompanied by a reduction in overt spongiosis. These chronic changes can partially obscure classical features and complicate histopathological diagnosis.

Schematic comparison of canonical immune polarization and histopathological features of atopic dermatitis (AD) and psoriasis. AD is traditionally characterized by spongiotic dermatitis with dominant Type 2 inflammation (Th2-driven; IL-4, IL-13, IL-5), barrier dysfunction, eosinophilic infiltration, and IgE responses. In contrast, psoriasis shows psoriasiform hyperplasia with Th1/Th17 axis (IL-17A/F, IL-22), epidermal hyperplasia, parakeratosis, diminished granular layer, and neutrophil recruitment. This binary framework has guided disease classification and targeted therapies, yet increasingly fails to account for mixed phenotypes, population variants, and therapy-induced shifts.

### 3.2 Dominant immune pathways

Immunologically, AD has historically been defined as a canonical type 2–mediated inflammatory disease. Th2 cells and their signature cytokines—interleukin (IL)-4, IL-13, and IL-5—play central roles in the initiation and maintenance of the disease. These cytokines compromise the epidermal barrier by downregulating filaggrin and lipid synthesis, while simultaneously promoting IgE class switching and eosinophil recruitment (17, 18). This immune-stromal crosstalk is amplified by keratinocytes, which act as “signal transducers” by releasing alarmins such as thymic stromal lymphopoietin (TSLP), IL-33, and IL-25 to enforce Th2 polarization (19, 20).

In chronic lesions, the immune landscape extends beyond the Th2 axis. Th22 cells and IL-22 upregulation become prominent, driving the epidermal hyperplasia (acanthosis) that typifies lichenification (21). Furthermore, TRMs accumulate in resolved lesions, contributing to the “molecular scar” that promotes site-specific recurrence (22).

Crucially, the immune architecture of AD is not uniform. This heterogeneity is evident in the “Asian phenotype” of AD, which contrasts with the predominantly Th2-driven profile in European populations. Asian patients frequently exhibit a potent Th17 axis activation alongside the classic Th2 signature, resulting in well-demarcated, psoriasiform plaques with parakeratosis, blurring the boundaries between AD and psoriasis (5, 23). This distinct immunophenotype challenges the “one-size-fits-all” model and underscores why certain populations show differential responses to biologics targeting the Th2 pathway (24, 25).

### 3.3 Controversies and heterogeneity

Despite advances in mapping the AD cytokine network, significant controversies remain regarding disease classification and evolution. A central debate revolves around the relative contribution of non-Th2 pathways (Th1/Th17) versus the concept that AD is fundamentally a Th2-driven disorder with secondary inflammation (17, 26, 27). Pathological interpretation is further complicated by the dynamic nature of the disease, particularly the “acute-on-chronic” phenomenon. Rather than a binary between acute spongiosis and chronic lichenification, patients frequently present with acute inflammatory flares superimposed upon established, remodeled tissue. In these “acute-on-chronic” lesions, the immune signature is complex, featuring a simultaneous surge of rapid-acting inflammatory cytokines against a background of structural fibrosis and epidermal remodeling (28). This dynamic progression complicates histopathological diagnosis, as a single biopsy may only represent a snapshot of a shifting immune-pathological continuum (29).

The era of targeted therapy has revealed latent immune plasticity, with therapies such as dupilumab—an IL-4 receptor antagonist—sometimes unmasking a psoriasiform phenotype, a phenomenon often referred to as a paradoxical reaction (30). This suggests the presence of repressive immune mechanisms between pathways, indicating that AD is not a static disorder but rather a dominant state within a fluid spectrum of cutaneous inflammation (20). Thus, recognizing the heterogeneity of AD, from the Asian phenotype to acute-on-chronic dynamics, is crucial for refining diagnostic criteria and precision therapeutic strategies (31–33).

## 4 Psoriasiform dermatoses: immune activation and tissue hyperplasia

### 4.1 Hallmark histopathological changes

The defining histopathological feature of psoriasiform dermatoses is regular acanthosis, characterized by the uniform elongation of rete ridges. This is often associated with thinning of the suprapapillary plates, parakeratosis (retention of nuclei in the stratum corneum), and a diminished or absent granular layer, reflecting accelerated keratinocyte proliferation and impaired terminal differentiation (**Figure 1**) (34, 35).

The inflammatory infiltrate in psoriasiform dermatoses is distinct. Neutrophils migrate through the epidermis, forming Munro’s microabscesses in the stratum corneum or spongiform pustules of Kogoj in the spinous layer. The underlying papillary dermis exhibits dilated, tortuous capillaries, which facilitate leukocyte trafficking and contribute to the clinical sign of Auspitz bleeding (36). A mixed infiltrate of T lymphocytes, dendritic cells, and macrophages typically surrounds these vessels. The intensity of these features varies depending on the disease stage and treatment, contributing to histological heterogeneity (37).

### 4.2 IL-23/Th17 axis and beyond

The IL-23/Th17 axis is the central molecular driver of psoriasiform inflammation. Activated dendritic cells release IL-23, which sustains Th17 cells and other IL-17–producing populations, including γδT cells. The effector cytokines IL-17A, IL-17F, and IL-22 act directly on keratinocytes to promote the hyperproliferative phenotype observed histologically. Specifically, IL-22 induces keratinocyte stemness and hyperplasia, while IL-17 facilitates neutrophil recruitment (38, 39).

However, the immune landscape is more complex. TRMs are enriched in resolved lesions and are implicated in the “molecular memory” that drives rapid recurrence at the same anatomical sites (40). Recent evidence also highlights that psoriasiform dermatoses are not exclusively Th17-driven; crosstalk with Th1 pathways (IFN-γ), innate immune sensors, and metabolic factors adds complexity, suggesting that the IL-23/Th17 axis is a dominant but not exclusive conductor of the inflammatory process (41, 42).

### 4.3 Psoriasiform reaction pattern vs. disease entity

It is essential to distinguish between psoriasis as a systemic inflammatory disease and psoriasiform dermatitis as a morphological reaction pattern. Psoriasiform dermatitis can be triggered by various stimuli, independent of genetic predisposition to psoriasis (43, 44). A striking example is drug-induced psoriasiform eruptions, such as those seen with anti-TNFα agents used in Crohn’s disease or rheumatoid arthritis. TNF blockade can disrupt the local cytokine balance, leading to an unimpeded type I interferon response that drives IL-23/Th17 activation, resulting in *de novo* psoriasiform eruptions in patients with no prior skin disease (45, 46).

This phenomenon highlights the immunological plasticity of the skin, which can undergo “phenotypic drift” under selective pressure. Consequently, recognizing psoriasiform inflammation as a shared tissue response—rather than merely a manifestation of psoriasis vulgaris—is vital. This underscores the importance of interpreting histopathology in the context of clinical history and treatment exposure, particularly in an era where immunomodulatory therapies often induce such pathological shifts (47, 48).

## 5 Immunological overlap, cellular plasticity, and diagnostic challenges

While AD and Psoriasis have traditionally been conceptualized as distinct entities defined by divergent immune polarizations—Th2 versus IL-23/Th17—emerging evidence suggests these boundaries are far more permeable than previously thought (49, 50). This overlap is not merely a confusion of symptoms but reflects the inherent immunological plasticity of the cutaneous immune system (51). The immune–pathological overlap and therapeutic implications are further integrated in **Supplementary Table 1**.

### 5.1 Cellular plasticity and lineage flexibility

**Figure 2:**
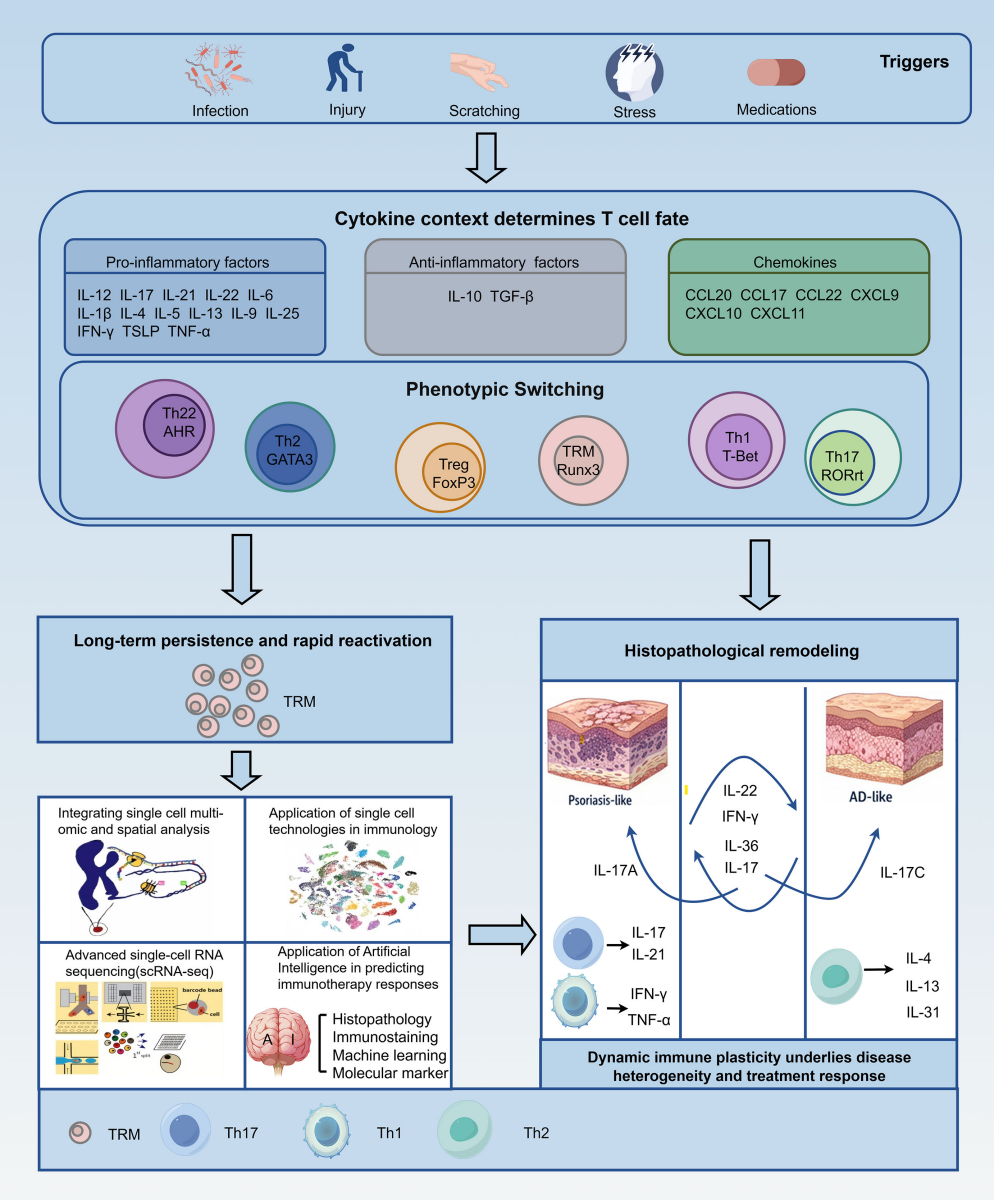
Dynamic immune plasticity drives disease heterogeneity and treatment responses in inflammatory skin diseases.

At the cellular level, the concept of rigid “lineage commitment” is being replaced by a model of functional flexibility (52). T helper cells retain the capacity to reprogram their phenotype in response to the changing cytokine milieu (53). For instance, Th17 cells exhibit significant instability; depending on environmental cues (e.g., the presence of TGF-β or IL-23), they can transdifferentiate into anti-inflammatory, IL-10-producing Tr1-like cells (regulatory plasticity) or shift towards a pathogenic IFN-γ-producing Th1-like phenotype (54) (**Figure 2**).

Furthermore, “hybrid” cell populations have been identified. In chronic AD lesions and Asian phenotypes, researchers have detected Th2/Th17 double-positive cells that simultaneously express GATA3 and RORγt (6, 55, 56). These hybrid cells embody the physical merger of two pathways previously considered mutually exclusive, explaining why some patients exhibit mixed histological features that defy binary classification (57).

This conceptual model shows how environmental triggers (e.g., infection, injury, stress, or drugs) disrupt cutaneous immune homeostasis, triggering cytokine-driven reprogramming of T helper cells. Rather than rigid lineages, effector T cells exhibit flexibility, with transitions and coexistence of Th2, Th17, Th22, and Th1 programs based on local cytokine milieu. Tissue-resident memory T cells (TRM) persist in resolved skin as a “molecular scar,” enabling rapid, phenotype-dependent reactivation. Selective therapies may rebalance or destabilize this network, leading to phenotypic drift, overlap syndromes, or paradoxical reactions. Thus, atopic dermatitis and psoriasis-like conditions form dominant states along a continuous immune-pathological spectrum, not discrete entities.

### 5.2 The temporal plasticity of TRM cells

Plasticity is also evident in the temporal dimension, primarily mediated by TRM cells (58). TRM cells persist in clinically resolved skin, functioning as a “molecular scar” (59). Their plasticity lies in their ability to dynamically switch functional states: remaining quiescent for surveillance during remission, yet rapidly reactivating into a potent effector state upon antigen re-exposure (60). This state-switching capability drives *in situ* recurrence, explaining why lesions tend to recur in the exact same anatomical locations (61). In the context of overlap, an AD-derived TRM pool might persist even as a patient develops psoriasiform features, creating a complex “layered” immune memory that complicates treatment (62).

### 5.3 Diagnostic and therapeutic implications

This cellular malleability has profound clinical implications (63). The suppression of a dominant pathway can disrupt the immunological equilibrium, allowing a suppressed sub-population to expand (64). A prime example is the unmasking of latent Th17 responses following aggressive Th2 blockade, leading to paradoxical psoriasiform eruptions (65, 66).

Consequently, traditional histopathological diagnosis based on static “snapshots” faces significant challenges (67). Single-lesion biopsies may fail to capture the dynamic evolution of the infiltrate (6). To address this, clinicians and pathologists must move towards a spectrum-based approach, integrating clinical history, anatomical distribution, and potentially molecular profiling (68, 69). Understanding these plasticity mechanisms is crucial not only for accurate diagnosis but also for anticipating therapeutic shifts, guiding the selection of biologics that modulate the immune network rather than merely suppressing a single node (70).

## 6 Discussion

The preceding sections highlight the complex interplay between immune dysregulation and pathological remodeling in AD and psoriasiform dermatoses. While considerable progress has been made in elucidating cellular networks, cytokine pathways, and histopathological correlates, several critical gaps remain, limiting the full translational potential of current knowledge.

### 6.1 Current research gaps

Traditional histopathological assessment provides a snapshot of tissue architecture at a single time point, which may not fully capture the dynamic nature of immune activation, cytokine flux, or cellular trafficking. This disconnect limits the ability to infer disease trajectory or predict treatment response based solely on morphology (71, 72). TRMs have emerged as central mediators of localized, site-specific relapse in both AD and psoriasiform dermatoses. However, their long-term persistence, functional plasticity, and interactions with other immune and structural cells remain incompletely understood, and standardized approaches to measure or modulate TRM activity are lacking (73–75). Despite advances in targeted biologics and small molecules, histopathological subtyping has not yet been systematically incorporated into treatment decision-making. Mixed or evolving reaction patterns further complicate the predictive value of static histology, underscoring the need for integrated immune–pathological frameworks (76, 77).

### 6.2 Translational implications

Integrating histopathological features with immune profiling can identify dominant inflammatory axes (e.g., Th2 vs Th17/Th22) within individual lesions, providing a rational basis for selecting targeted therapies. Such approaches may improve response rates, reduce adverse effects, and minimize trial-and-error prescribing in complex or mixed-pattern cases (78, 79). The advent of highly selective immunotherapies has revealed previously unrecognized immune plasticity, including shifts in inflammatory programs under cytokine blockade (80). This underscores the need for pathologists to move beyond descriptive histology toward functional interpretation, integrating cellular, molecular, and spatial data to guide clinical management and monitor therapy-induced tissue remodeling (81).

### 6.3 Future developments

The future of characterizing inflammatory skin diseases lies in the convergence of high-dimensional molecular profiling and computational analysis. Spatial transcriptomics and multiplex immunofluorescence now allow for the simultaneous mapping of cell types, cytokine expression, and signaling pathways while preserving the native tissue architecture. Applying these tools can elucidate local immune niches—such as the “synapse” between nerve fibers and immune cells—providing mechanistic insights that bulk sequencing cannot reveal (82–84).

Complementing these molecular tools, Artificial Intelligence (AI) and computational pathology are emerging as transformative assets. Deep learning algorithms can be trained to recognize complex, “sub-visual” morphological features and immune infiltration patterns that are subtle or imperceptible to the human eye. This is particularly critical for the “grey zone” cases, such as distinguishing atypical Asian AD from psoriasiform dermatitis, where conventional microscopy may be inconclusive. AI models can provide objective, quantitative scoring of histological probability, serving as a powerful decision-support tool for pathologists (85–88).

Moving forward, the field must transition from rigid categorical diagnoses toward dynamic immune–pathological profiling. By synthesizing morphological data with spatial-molecular and AI-driven insights, pathology can evolve from a primarily diagnostic discipline into a central component of precision medicine. This expanded role encompasses risk stratification, prediction of therapeutic response (e.g., identifying patients likely to experience paradoxical reactions), and longitudinal monitoring of disease evolution (89, 90).

In summary, recent advances in understanding the immunopathogenesis of inflammatory skin diseases, particularly psoriasis (PsO) and atopic dermatitis (AD), have shifted the conceptual paradigm from viewing these conditions primarily as static pathological states—characterized by fixed histological features such as epidermal hyperplasia in PsO or spongiosis in AD—to recognizing them as highly dynamic immune processes. This transition is vividly illustrated in the updated schematic (**Figure 2**), which integrates key histological and immunological elements, including cytokine axes (e.g., IL-17/IL-23 in PsO and type 2 cytokines in AD), the persistence and reactivation of tissue-resident memory T cells (Trm), and pathway plasticity, while employing distinct visual coding (blue tones for AD-dominant features and red tones for PsO) to highlight bidirectional influences and convergence under targeted therapies (91, 92).

Cutting-edge technologies, including single-cell RNA sequencing (scRNA-seq), spatial transcriptomics, high-resolution multi-omics analyses, and emerging artificial intelligence applications for immune phenotype prediction, have significantly deepened insights into immune plasticity, heterogeneous cellular states (e.g., inflammatory fibroblasts responsive to IL-23 blockade), treatment-induced reprogramming (such as post-dupilumab changes in Trm populations), paradoxical reactions, phenotype switching, and disease heterogeneity across PsO and AD (93–95).

Looking ahead, future research should prioritize longitudinal multi-omics studies integrating scRNA-seq with spatial transcriptomics to map dynamic immune reprogramming at single-cell resolution during and after therapy, elucidate the mechanisms underlying Trm persistence and reactivation in non-lesional/healed skin, investigate AI-driven predictive models for personalized treatment responses and phenotype switching, and explore cross-disease convergences and divergences to develop more precise, durable interventions that target not only active inflammation but also the residual immunological memory that perpetuates these chronic conditions (96–100).

## 7 Conclusion

Atopic dermatitis and psoriasiform dermatoses exemplify the dynamic interplay between immune dysregulation and structural skin remodeling. Although traditionally viewed as separate conditions, growing evidence reveals a disease continuum characterized by significant phenotypic overlap, immunological plasticity, and heterogeneity. Consequently, dependence on static histological features is increasingly inadequate.

Combining morphological analysis with dynamic immune profiling provides a powerful framework for deciphering disease mechanisms and predicting treatment responses, including potential phenotype shifts. Driven by advances in spatial biology and AI-assisted diagnostics, this integrative approach is poised to redefine disease classification. It elevates pathology from a mere diagnostic endpoint to a cornerstone of precision medicine, enabling the development of adaptable, personalized treatment strategies for complex skin diseases.
