# Supplementary material for: From static pathology to dynamic immunity: immunological plasticity and histopathological remodeling in atopic dermatitis and psoriasis
Source: Front Immunol. 2026 Feb 13;17:1770896. doi: 10.3389/fimmu.2026.1770896 (PMC12945763; doi:10.3389/fimmu.2026.1770896)
Supplement: Supplementary file 1 [file Table1.docx]

**Supplementary Table 1 Comparative immune–pathological features of atopic dermatitis, psoriasiform dermatoses, and overlap phenotypes**

|  | **Atopic Dermatitis (AD)** | **Psoriasiform Dermatoses / Psoriasis** | **Overlap / Plasticity States** |
| --- | --- | --- | --- |
| **Main histopathology** | Spongiosis, lymphocyte exocytosis; chronic lesions with lichenification | Regular acanthosis, parakeratosis, diminished granular layer | Mixed spongiotic–psoriasiform patterns; stage- and site-dependent [1,2] |
| **Key epidermal changes** | Barrier dysfunction; variable hyperplasia | Accelerated keratinocyte turnover; suprapapillary thinning | Dynamic remodeling; hyperplasia with residual spongiosis [3-5] |
| **Key immune axis** | Th2 (IL-4, IL-13, IL-5); Th22 in chronic AD | IL-23/Th17 (IL-17A/F, IL-22); Th1 contribution [3-5] | Co-activation of Th2 and Th17 pathways |
| **Characteristic immune cells** | Th2, ILC2, eosinophils, mast cells | Th17, γδT cells, neutrophils, dendritic cells | Hybrid Th2/Th17 cells; context-dependent effector skewing |
| **TRM features** | Persistent Th2-biased TRM driving site-specific relapse | IL-17/IL-22-competent TRM mediating molecular memory [6-7] | Layered TRM pools with functional reprogramming |
| **Typical cytokine milieu** | TSLP, IL-33, IL-4/13 | IL-23, IL-17, IL-22, IFN-γ | Cytokine balance shifts under environmental or therapeutic pressure |
| **Clinical phenotype** | Eczematous, pruritic, often diffuse | Well-demarcated plaques, scaling | Asian AD phenotype; paradoxical eruptions |
| **Response to targeted therapy** | Strong response to anti-IL-4/13; paradoxical psoriasis possible [2] | Robust response to anti-IL-23/IL-17 [2,10] | Variable: pathway blockade may unmask alternative inflammation [2,10] |
| **Interpretation** | Dominant state within inflammatory spectrum [8-9] | Another dominant state within spectrum | Transitional or unstable immune-pathological state [9] |

**References:**

1. Guttman-Yassky E, Krueger JG. Atopic dermatitis and psoriasis: two different immune diseases or one spectrum? Curr Opin Immunol. 2017 Oct;48:68-73. doi: 10.1016/j.coi.2017.08.008. Epub 2017 Sep 1. PMID: 28869867.

2. Noda S, Krueger JG, Guttman-Yassky E. The translational revolution and use of biologics in patients with inflammatory skin diseases. J Allergy Clin Immunol. 2015 Feb;135(2):324-36. doi: 10.1016/j.jaci.2014.11.015. Epub 2014 Dec 23. PMID: 25541257.

3. Noda S, Suárez-Fariñas M, Ungar B, Kim SJ, de Guzman Strong C, Xu H, Peng X, Estrada YD, Nakajima S, Honda T, Shin JU, Lee H, Krueger JG, Lee KH, Kabashima K, Guttman-Yassky E. The Asian atopic dermatitis phenotype combines features of atopic dermatitis and psoriasis with increased TH17 polarization. J Allergy Clin Immunol. 2015 Nov;136(5):1254-64. doi: 10.1016/j.jaci.2015.08.015. Epub 2015 Oct 1. PMID: 26428954.

4. Lowes MA, Suárez-Fariñas M, Krueger JG. Immunology of psoriasis. Annu Rev Immunol. 2014;32:227-55. doi: 10.1146/annurev-immunol-032713-120225. PMID: 24655295; PMCID: PMC4229247.

5. Nestle FO, Kaplan DH, Barker J. Psoriasis. N Engl J Med. 2009 Jul 30;361(5):496-509. doi: 10.1056/NEJMra0804595. PMID: 19641206.

6. Clark RA. Resident memory T cells in human health and disease. Sci Transl Med. 2015 Jan 7;7(269):269rv1. doi: 10.1126/scitranslmed.3010641. PMID: 25568072; PMCID: PMC4425129.

7. Cheuk S, Schlums H, Gallais Sérézal I, Martini E, Chiang SC, Marquardt N, Gibbs A, Detlofsson E, Introini A, Forkel M, Höög C, Tjernlund A, Michaëlsson J, Folkersen L, Mjösberg J, Blomqvist L, Ehrström M, Ståhle M, Bryceson YT, Eidsmo L. CD49a Expression Defines Tissue-Resident CD8+ T Cells Poised for Cytotoxic Function in Human Skin. Immunity. 2017 Feb 21;46(2):287-300. doi: 10.1016/j.immuni.2017.01.009. Epub 2017 Feb 14. PMID: 28214226; PMCID: PMC5337619.

8. Eyerich K, Eyerich S. Immune response patterns in non-communicable inflammatory skin diseases. J Eur Acad Dermatol Venereol. 2018 May;32(5):692-703. doi: 10.1111/jdv.14673. Epub 2018 Jan 15. PMID: 29114938; PMCID: PMC5947562.

9. O'Shea JJ, Paul WE. Mechanisms underlying lineage commitment and plasticity of helper CD4+ T cells. Science. 2010 Feb 26;327(5969):1098-102. doi: 10.1126/science.1178334. PMID: 20185720; PMCID: PMC2997673.

10. Conrad C, Gilliet M. Psoriasis: from Pathogenesis to Targeted Therapies. Clin Rev Allergy Immunol. 2018 Feb;54(1):102-113. doi: 10.1007/s12016-018-8668-1. PMID: 29349534.
